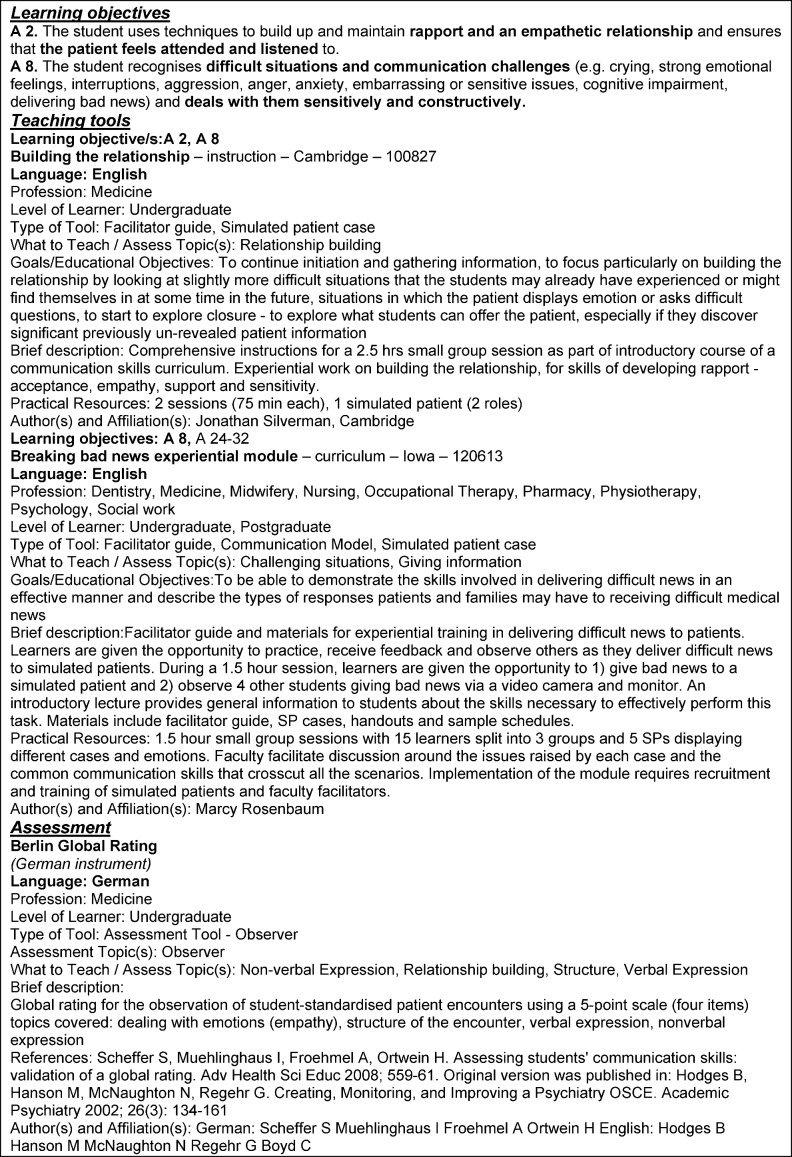# Communication in Health Professions: A European consensus on inter- and multi-professional learning objectives in German

**DOI:** 10.3205/zma001022

**Published:** 2016-04-29

**Authors:** Cadja Bachmann, Claudia Kiessling, Anja Härtl, Rainer Haak

**Affiliations:** 1 Universitätsklinikum Hamburg-Eppendorf, Institut für Allgemeinmedizin, Hamburg, Germany; 2 Medizinische Hochschule Brandenburg Theodor Fontane, Neuruppin, Germany; 3 Klinikum der Universität München, Institut für Didaktik und Ausbildungsforschung in der Medizin, München, Germany; 4 Universitätsklinikum Leipzig, Poliklinik für Zahnerhaltung und Parodontologie, Leipzig, Germany

**Keywords:** interprofessional, multiprofessional, core communication curriculum, health care professions, communication curriculum for all health care professions

## Abstract

**Background and aim: **Communication is object of increasing attention in the health professions. Teaching communication competencies should already begin in undergraduate education or pre-registration training.

The aim of this project was to translate the Health Professions Core Communication Curriculum (HPCCC), an English catalogue of learning objectives, into German to make its content widely accessible in the German-speaking countries. This catalogue lists 61 educational objectives and was agreed on by 121 international communication experts. A European reference framework for inter- and multi-professional curriculum development for communication in the health professions in German-speaking countries should be provided.

**Method: **The German version of the HPCCC was drafted by six academics and went through multiple revisions until consensus was reached. The learning objectives were paired with appropriate teaching and assessment tools drawn from the database of the teaching Committee of the European Association for Communication Health Care (tEACH).

**Results: **The HPCCC learning objectives are now available in German and can be applied for curriculum planning and development in the different German-speaking health professions, the educational objectives can also be used for inter-professional purposes. Examples for teaching methods and assessment tools are given for using and implementing the objectives.

**Conclusion: **The German version of the HPCCC with learning objectives for communication in health professions can contribute significantly to inter- and multi-professional curriculum development in the health care professions in the German-speaking countries. Examples for teaching methods and assessment tools from the materials compiled by tEACH supplement the curricular content and provide suggestions for practical implementation of the learning objectives in teaching and assessment. The relevance of the German HPCCC to the processes of curriculum development for the various health professions and inter-professional approaches should be the subject of further evaluation.

## Zusammenfassung

**Hintergrund und Zielsetzung: **Kommunikation rückt in den Gesundheitsberufen zunehmend in den Blickpunkt. Die Vermittlung kommunikativer Kompetenzen sollte bereits im Studium bzw. in der Ausbildung beginnen.

Ziel des Projekts war, einen englischsprachigen Ausbildungszielkatalog für die Kommunikation in den Gesundheitsberufen, das „Health Professions Core Communication Curriculum (HPCCC)“, ins Deutsche zu übersetzen und einer breiten deutschsprachigen Öffentlichkeit zugänglich zu machen. Der Katalog umfasst 61 Ausbildungsziele und wurde von 121 internationalen Kommunikationsexperten konsentiert. Ein europäischer Bezugsrahmen für die inter- und multiprofessionelle Curriculumentwicklung „Kommunikation in den Gesundheitsberufen“ im deutschsprachigen Raum sollte hergestellt werden.

**Methode:** Die deutschsprachige Version des HPCCC wurde von sechs Personen entwickelt und in mehreren Revisionsschleifen konsentiert. Beispielhaft wurden Ausbildungsziele mit geeigneten didaktischen Methoden und Prüfungsinstrumenten aus der Materialiensammlung des teaching committee of the European Association for Communication Health Care (tEACH) versehen.

**Ergebnisse: **Die Ausbildungsziele des HPCCC liegen nun in deutscher Version vor und können für die Curriculumsplanung und -entwicklung in den einzelnen deutschsprachigen Gesundheitsberufen aber auch berufsübergreifend genutzt werden. Anwendungsbeispiele für didaktische Methoden und Prüfungsinstrumente werden aufgelistet und zeigen konkrete Umsetzungsmöglichkeiten auf.

**Fazit: **Die deutschsprachige Version des HPCCC, die Ausbildungsziele für die Kommunikation in den Gesundheitsberufen aufzeigt, kann einen wichtigen Beitrag zur inter- und multiprofessionellen Curriculumentwicklung in den Gesundheitsberufen im deutschen Sprachraum leisten. Beispiele für didaktische Methoden und Prüfungen aus der Materialiensammlung der tEACH ergänzen das curriculare Konzept und sollen Anregungen für die praktische Umsetzung der Ausbildungsziele in der Lehre und in Prüfungen geben. Die Bedeutung des deutschsprachigen HPCCC auf die curricularen Entwicklungsprozesse in den verschiedenen Gesundheitsberufen und für interprofessionelle Ansätze bedarf künftig weiterer Evaluationen.

## Background and aim

Communication in the health care professions has gained in importance over recent years in respect to education and advanced professional training [1], [2], [3], [4], [5], [6]. Scientific studies have demonstrated that skilled communication has positive effects on patients´ satisfaction, adherence to therapy and treatment outcomes [7], [8], [9], [10], [11], [12]. Accordingly, communication skills should be taught during undergraduate education or training in the health professions. Numerous recommendations exist for medical education defining the core communication competencies for physicians-to-be [13], [14], [15], [16], [17], [18]. An inter- or multi-professional approach to imparting communication skills during training in the health professions did not exist.

To address this issue, a multi-professional European working group of the teaching Committee of the European Association for Communication in Healthcare (tEACH) developed an English-speaking catalogue of learning objectives for a core communication curriculum that can be applied, both inter- and multi-professionally, to all the health professions [19]. The Health Professions Core Communication Curriculum (HPCCC) was developed on basis of the Basler Consensus Statement, which was published in 2008 by the GMA committee on communication and social skills in undergraduate medical education [16].

The HPCCC defines 61 learning objectives as core communication skills for all health professions. In a multi-step consensus process, the HPCCC was agreed upon by 121 communication experts. The experts had on average been active in communication education for 12.4 years. The experts represented 16 European countries and 15 health professions, i.e. medicine, nursing, dentistry, pharmacy, psychology, physiotherapy, occupational therapy or speech- and- language therapy. Around 40% were involved in medical education and advanced professional training, and nearly 60% in other health professions. Many of them taught in more than one health profession (i.e. medical and nursing education). The acceptance of the educational objectives was very high, ranging between 84-100%. In 2013 the HPCCC was published in English [19]. The method for the development of the learning objectives, the consensus process, and the relevance of the learning objectives are described in detail in the original article.

Based on the broad expert consensus, the HPCCC can serve as a guide for curriculum development for the health professions and be used to evaluate existing communication curricula. It has been designed as a flexible framework and can be integrated into health profession curricula in parts or in total. The learning objectives are defined for inter-professional (across health professions) and for multi-professional (different health professions) purposes.

Since the HPCCC has until now only been available in English, the German working group has translated the educational objectives aiming to make them more widely accessible in the German-speaking countries.

## Method

The English version of the HPCCC was translated and edited by six members of the working group. Three of the authors (CB, CK and RH) are members of the tEACH committee. Two medical doctors (CB, AH) and two medical students (KB, MTS) independently translated the HPCCC. The goal of translation was to remain very close to the original version, while adapting the content to German language. The drafts of the initial translations were compiled into a rough draft, which was then reviewed by two other authors (CK, RH) to check for accuracy of content and understandability. Minor corrections were made to improve fluency. Adaptions for gender were undertaken in most cases, but left in others to maintain readability. If a masculine or feminine formulation is used, the opposite gender is always automatically included in the meaning.

A final draft was agreed upon after several discussions and revisions and a consensus regarding the final German translation was reached. Since two of the authors (CB, RH) participated in development process of the original version of the HPCCC and were familiar with the educational objectives, retranslation into English was not undertaken.

In addition to the German translation, examples of internationally established teaching methods and adequate assessment tools were paired with individual learning objectives to offer concrete examples for practical implementation. The materials compiled by tEACH—encompassing more than 100 teaching tools and more than 30 mostly validated assessment tools—were reviewed for this purpose. The collection is available on the website of the tEACH committee of the European Association for Communication in Healthcare [http://www.each.eu]. These materials can be accessed through a database and are mainly available in English. However, German examples are also present.

## Results

The educational objectives of the HPCCC are now available in German and can be used inter- and multi-professionally by curriculum designers and teachers for the various health professions in the German-speaking countries. Thus, the requirement for a German version, in particular by the non-medical professions, has been covered.

### Learning objectives

Educational objectives were defined as objectives that students should have accomplished at graduation or pre-registration. The 61 learning objectives of the HPCCC are grouped under three topics:

A) Communication with patients (n=34 objectives)

B) Reflection and professionalism (n=12 objectives)

C) Communication in health care teams (n=15 objectives)

The learning objectives are sequenced in overarching and specific goals (see Attachment 1).

The highest level of acceptance among the international communication experts was reached for the learning objectives in topic A regarding the numbers 1, 2, 4, 6, 8, 9, 12, 14, 17, 19 and 25. 100% of the experts rated these objectives as most or very important. For 45 other learning objectives the degree of acceptance was between 90-99%; for five others, acceptance was between 84-89%. The original article lists the importance of each learning objective based on means and acceptance. Qualitative analyses provide further insights into the relevance of the HPCCC [19].

#### Teaching and assessment

Appropriate teaching methods were assigned to the HPCCC learning objectives. The assessment tools are designed to be more comprehensive and generally cover the assessment of overarching educational objectives. As examples for curriculum development, we selected specific learning objectives, appropriate teaching methods and assessment tools, which are presented in Figure 1 (Fig. 1). The teaching and assessment tools are presented in a brief description, more detailed materials are available in the tEACH database.

## Discussion

The English catalogue of learning objectives, the Health Professions Core Communication Curriculum (HPCCC), was developed with regard to the increasing interest of teaching communication skills in the health professions. A total of 121 international communication experts from many different health professions have reached a consensus on this catalogue [19]. The high acceptance of the learning objectives among experts indicates the relevance of this tool. The HPCCC is the worldwide broadest consensus on inter-professional communication learning objectives for the health professions.

Based on the broad and international level of consensus with experts from various health care professions, the HPCCC is obviously suitable for communication curriculum development in the individual health professions. Hence, practicability and implementation of the objectives are currently evaluated in various European countries (i.e. UK, Germany, Poland, Portugal) and health professions (i.e. medicine, nursing, pharmacy). The first evaluations, not yet published, are positive.

Within this context and with the intent of making the catalogue more widely available to audiences in the German-speaking countries, the HPCCC was translated into German. The HPCCC is now available as such and can give significant contribution to the development of comprehensive communication curricula in many German-speaking health professions. The HPCCC can be used by the different professions, ranging from medicine to nursing to other therapeutic occupations, i.e. physiotherapy, and for inter-professional education. In particular, those health professions with no or few initiatives for structured teaching of communication can benefit from this catalogue.

In addition, the HPCC has been enhanced by appropriate teaching and assessment tools. The tEACH database offers numerous internationally applied tools for both areas. More than 100 teaching examples have been matched to the HPCCC learning objectives. There is also an amount of materials that can be used in the inter-professional context, along with more than 30 mainly validated assessment tools. The assessment tools are in general designed for rather holistic approaches and rather address the overarching educational objectives of the HPCCC. The tEACH materials are predominantly in English, but there are some German documents available. The documents are freely accessible or available in the EACH membership area and can be viewed and downloaded. The HPCCC learning objectives combined with tEACH’s various teaching and assessment materials allow curriculum planners sufficient scope for developing or restructuring communication curricula.

The flexibility of the HPCCC in terms of inter- and multi-professional use is an advantage. The learning objectives are suitable not only for multi-professional concepts, meaning those for individual health professions, but also for inter-professional approaches across different occupational groups. For this approach the existing model offers an additional contribution to the recommendations given in the position paper of the GMA committee on inter-professional education in the health professions [20]. As a result, the German version of the HPCCC can also serve as a contribution to further developments in teaching inter-professional communication in the different health professions in German-speaking countries.

The HPCCC learning objectives for communication were developed on the Basler Consensus Statement [16] and on other consensus statements [13], [14], [15], [17], [18], as well as 25 other articles listed in the original paper [19]. In contrast to the existing consensus statements which focus on medical education, the HPCCC was designed for education in communication in all the health professions and thus follows a multi- and inter-professional approach. Hence, discussions referring the development of medical education can also benefit from the HPCCC, since inter-professional perspectives on the development and implementation of communication curricula still often go largely unheeded. 

The importance of the HPCCC for curriculum development in the health professions in the German-speaking countries cannot be estimated at this point in time. The degree to which implementation of the German HPCCC in the health professions is successful and being used to shape inter-professional teaching concepts awaits to be seen and offers an interesting option for curriculum planning and teaching in the German-speaking countries. The authors welcome feedback on the applicability and practicability of the German HPCCC, as well as the opportunity to engage in further dialogue on these topics.

## Limitations

The aim of the HPCCC was to provide communication learning objectives for undergraduate education which are important for all health care professions and in addition to harmonize the content of core communication skills within the different occupational groups. Communication learning objectives specific to particular professions were intentionally not included. This primarily involves the topic of treatment and therapy planning. To address this aspect, the various health professions are requested to develop their own approaches and educational objectives to supplement this core curriculum with additional specific communication learning objectives. 

The learning objectives were developed and agreed upon by communication experts with many years of experience in communication skills teaching and assessment. Consensus statements and the results published in the literature, which not only touch upon patient perspectives and therapy success but also the perspectives of those health professions that have previously been less strongly represented than the medical professions, have played an important role in the development process.

Experts were involved in the first phase of reaching a consensus. Next steps intend to continue the evaluation of the acceptance of the HPCCC by the different health professions, patients, students and instructors.

## Practice implications

The HPCCC is currently evaluated in several countries with regard to education in different health professions. The preliminary results, yet to be published, are positive. The non-medical health professions benefit particularly from this tool, since no comparable consensus statement on educational objectives concerning communication competencies currently exists for these occupational groups.

This guide already finds application in German medical education but it is also used in countries where no national recommendations have been made to date. The different health professions use the HPCCC to design new communication curricula or for curriculum mapping, meaning the comparison of currently implemented learning objectives with the recommendations. The first inter-professional concepts and approaches are currently developed.

Recommendations for medical education in Germany and in the other German-speaking countries exist. Along with the Basler Consensus Statement and the longitudinal model curriculum [16], [17], the national catalogues of competency-based learning objectives for undergraduate medical and dental education were published in 2015 [http://www.nklm.de], [http://www.nklz.de]. The HPCCC represents an addition to the existing models. However, it goes a step beyond with its inter- and multi-professional approach to provide learning objectives for all the health professions in regard to communication skills. With a total of 61 learning objectives, the HPCCC is a comparatively straightforward tool for curriculum development and can, in this context, also help to facilitate the processes of implementation and integration.

The tEACH database [http://www.EACH.eu] containing many examples of internationally applied teaching methods and assessment tools can serve as a helpful resource when implementing the HPCCC learning objectives. Not just curriculum designers and instructors, but also examiners, can make use of this extensive collection of materials to plan and prepare their own lessons or assessments. The database is constantly being improved and expanded in terms of user-friendliness and search options.

## Acknowledgement

We would like to thank Katharina Blum and Marie-Therese Suda, who helped draft the first version of the German HPCCC. In addition, we thank all the international communication experts who were involved in reaching the consensus on the English-language HPCCC. Furthermore, we wish to express our gratitude to all colleagues who shared their teaching and assessment tools and the tEACH working groups. Their long-term commitment and dedicated work has made the collection of extensive materials on teaching methods, assessment tools for curriculum development available. The resulting database represents a valuable resource for instructors and curriculum designers.

## Competing interests

The authors declare that they have no competing interests.

## Supplementary Material

learning objectives

## Figures and Tables

**Figure 1:**