# Supplementary material for: Communication in Health Professions: A European consensus on inter- and multi-professional learning objectives in German
Source: GMS J Med Educ. 2016 Apr 29;33(2):Doc23. doi: 10.3205/zma001022 (PMC4895849; doi:10.3205/zma001022)
Supplement: learning objectives [file JME-33-23-s-001.pdf]

**A. Communication with patients**	
Core communication objectives:	
	The student…
1	Adapts own communication to the **level of understanding and language** of the patient, avoiding jargon.
2	Uses techniques to build up and maintain **rapport and an empathetic relationship** and ensures that **the patient feels attended and listened** to.
3	Relates to the patient **respectfully** including ensuring confidentiality, privacy and autonomy and recognizes the **patient as a partner** in shaping a relationship.
4	Elicits and explores the **content** of the patient's bio-psycho-social history<br>(e.g. gathers relevant information, ensures understanding, relays information understandably, verbalises emotional content).
5	Encourages the patient to express own **ideas, concerns, expectations and feelings** and accepts **legitimacy of patient's views and feelings.**
6	**Gives information** to the patient (oral, written, electronic and over the phone) in a timely, comprehensive and meaningful manner.
7	Uses techniques of **active listening** (e.g. reflection, picking up patient´s cues, paraphrasing, summarising, verbal and non-verbal techniques).
8	Recognises **difficult situations and communication challenges** (e.g. crying, strong emotional feelings, interruptions, aggression, anger, anxiety, embarrassing or sensitive issues, cognitive impairment, delivering bad news) and **deals with them sensitively and constructively.**
9	Shows **awareness of the non-verbal communication of both** the patient and the healthcare professional (e.g. eye contact, gestures, facial expressions, posture) and responds to them appropriately.
10	Shapes a conversation from beginning to end with regard to **structure** (e.g. introduction, initiating the conversation, gathering and giving information, planning, closing interview, setting up next meeting; time management).
11	Uses different **types of questions** (e.g. open, closed and focused) according to the situation.
More detailed specific objectives, depending on context and situation:	
**Shaping of relationship:** *The student involves the patient in the interaction to establish a therapeutic relationship using a patient-centred approach*	
12	**Identifies patient expectations** with respect to the role of health care professional.
13	Uses adequate **strategies to solve conflicts** (e.g. feedback on perception, impact, wishes).
**Patient's perspective and health beliefs:** *The student orients own communication in line with the actual needs and concerns of the patient*	
14	Elicits the **needs and capabilities of the patient** (e.g information, autonomy, truth and responsibility) and **adapts the plan / intervention** to patient´s resources and strengths.
15	Considers **somatic, mental, social, gender, cultural, ethical and spiritual** elements in the care and assessment of the patient and perceives divergences between own values and norms and the patient's.
16	Responds to the **patient's health beliefs and theories of illness** and contrasts and integrates these into own theories of illness as a health care professional.
**Information:** *The student effectively collects and communicates relevant information for reasoning and decision-making*	
17	Finds out **how much information the patient requires** and **gives the appropriate amount of information.**
18	Provides **information in a patient-centered way** and shares it with the patient's consent (e.g. colleagues, family and others.)
19	Elicits and **synthesises information** for patient care.
20	Inquires about the **patient's level of knowledge** about the illness
21	Considers **different elements of a patient history** (history of the illness, history of the health care professional–patient relationship, history of the patient).
22	Knows about the importance of **supplementing verbal information** with diagrams, models, written information and instructions and applies the information appropriately.

23	Seeks out and synthesises relevant information **from other sources** (e.g. patient's family, caregivers and other professionals), if necessary and available
**Reasoning and decision-making:** *The student considers the extent to which individuals are involved and responsible in the reasoning and decision-making process*	
24	Ascertains **how much involvement and responsibility the patient** is willing and able to take for decision-making.
25	Discusses with the patient the likely **advantages, disadvantages and expected outcomes**
26	Encourages **active participation by** the patient **in decision-making** and **explains choices or rights** to the patient in a patient-centered manner.
27	Clarifies **own role in decision-making** process.
28	Discusses with patient the spectrum of possible **consequences of a decision** and explains to the patient the likely **consequences of not choosing diagnostic and therapeutic measures.**
29	Inquires about the **relevant psychological and social resources** the patient has available for making a decision.
30	Offers the patient the option to **include other people in the decision- making process** and clarifies with the patient how and when a decision must be made.
31	**Discusses decisions** with colleagues, patients and their relatives as appropriate and regularly **reassesses own decisions** and revises them if necessary.
32	Identifies **own opinion** clearly to the patient if asked.
**Uncertainty:** *The student respects uncertainty as an integral part of reasoning and decision-making*	
33	**Talks openly** to the patient about uncertainty and **formulates ways of dealing** with it.
34	Explains to the patient which **information is needed to minimize uncertainty** in the decision-making process.
**B. Intra- and interpersonal communication (Professionalism and Reflection)**	
**Communication and reflection with self and others**: *The student consistently develops and improves self-awareness, self-reflection, self-care and reflects with others on own communication and behaviour*	
1	**Recognises own emotions** (e.g. insecurity, sympathy/antipathy, attraction) in relation to others (e.g. patients, colleagues) and is able to **work efficiently despite own emotional reactions** when the situation requires to do so (e.g. degree of suffering of the patient, demanding patient).
2	Recognizes that effective communication with patients can **foster patients´ satisfaction and improved clinical outcomes**.
3	Describes and **assesses own communication and behaviour critically** considering alternatives in both.
4	Identifies, reflects and communicates **own strengths, weaknesses, limitations** and assesses **own wishes, fears, goals, norms and values.**
5	Assesses **own stereotypes and social prejudices** and is aware that own actions are influenced by personal experience, the current situation, own knowledge and interests.
6	Reflects on **own attitude towards work** (e.g. cynicism, satisfaction).
7	Reflects and discusses **ethical, inter-cultural and other challenges** in own actions as a health care professional (e.g. patient unable to give consent, health/illness concepts in different cultures, translators) and discusses approaches for resolution.
8	Uses authority and **influence responsibly.**
9	Analyses and **discusses conversational situations with others** (metacommunication in e.g. peer reflexion, team conferences, Balint groups).
10	Knows about **models of the health care professional–patient relationship** (e.g. transference/counter transference, reciprocity, inter-subjectivity, expectation/experience, potential imbalance of hierarchy).
**Dealing with errors and uncertainty:** *The student addresses errors and respects uncertainty as an integral part of reasoning and decision-making*	
11	**Addresses own and others errors appropriately** (e.g. refrains from personally allocating blame) in order to seek for solutions and assistance and knows about basic principles in the development of errors (e.g. neglecting information or patient´s needs, inadequate communication).
12	Addresses and **deals with own uncertainty** appropriate to own level of education.

**C. Communication in health care teams (Professional communication)**	
**Teamwork and professional communication:** *The student shows ability to communicate effectively in multi-professional teams*	
1	Perceives and **respects individuality, subjective perception, different points of view** of team members and the **expertise** of the different health care professionals.
2	Contributes to a **positive working atmosphere** (e.g. supports and integrates team members, mentions the positive side of unpleasant aspects, values team-success).
3	Uses **feedback** rules (e.g. first-person-statements) and gives feedback to team-members appropriately.
4	Is able to solve conflicts and enables a **constructive negotiation** in a healthcare team.
5	Takes on, clarifies and reflects on **own role and responsibilities in the team** (e.g. team vs. team-player, leader) and identifies the **role of own profession** in an inter-professional team.
6	Specifies and **appreciates own potential** with regard to the team and is **willing and able to work** with others.
7	Understands the **principles of team dynamics** and how factors both support and inhibit teamwork in practice**.**
8	Identifies **own interests** and distinguishes these from the team goals.
9	Reflects on the impact of own opinion **on others** and takes this into account.
**Leadership:** *The student shows basic competencies in leadership skills*	
10	Gives **clear instructions**.
11	Ensures that **all relevant information is available**.
12	Facilitates the formation of opinions in the group and encourages and rewards team members to **voice differing opinions**.
**Professional communication and management:** *The student uses effective and efficient communication and management strategies*	
13	Maintains **clear, appropriate records** (written or electronic) of clinical encounters and plans.
14	**Presents** expert knowledge **effectively** (e.g. presenting a patient and clinical details to others, speaking in front of a group, presenting scientific data).
15	Identifies and is knowledgeable of **how to refer** to people/ institutions/ agencies that can help to solve problems appropriate to the situation.
